# Endoscopic therapy replaces surgery for clinical T1 oesophageal cancer in the Netherlands: a nationwide population-based study

**DOI:** 10.1007/s00464-023-09914-x

**Published:** 2023-02-27

**Authors:** Irma C. Noordzij, Marije L. Hazen, Grard A. P. Nieuwenhuijzen, Rob H. A. Verhoeven, Erik J. Schoon

**Affiliations:** 1 Department of Gastroenterology and Hepatology, Catharina Hospital, Michelangelolaan 2, 5623 EJ, Postbus 1350, 5602 ZA Eindhoven, The Netherlands; 2 Department of Gastroenterology and Hepatology, Elkerliek Hospital, Postbus 98, 5700 Helmond, AB The Netherlands; 3 Department of Surgery, Catharina Hospital, Postbus 1350, 5602 Eindhoven, ZA The Netherlands; 4 Netherlands Comprehensive Cancer Organisation, Department of Research & Development, Postbus 19079, 3501 Utrecht, DB The Netherlands; 5 GROW School for Oncology and Reproduction, Maastricht University, Maastricht, The Netherlands

**Keywords:** Esophageal neoplasms, Esophageal squamous cell carcinoma, Esophageal adenocarcinoma, Surgery, Endoscopy, Survival rate

## Abstract

**Background:**

Endoscopic resection for early oesophageal cancer was introduced around 2000 in the Netherlands. The scientific question was how the treatment and survival of early oesophageal and gastro-oesophageal junction cancer has changed over time in the Netherlands.

**Methods:**

Data were obtained from the nationwide population-based Netherlands Cancer Registry. All patients diagnosed with clinical in situ or T1 oesophageal or GOJ cancer without lymph node or distance metastasis during the study period (2000–2014) were extracted. Primary outcome parameters were the trends in treatment modalities over time and relative survival of each treatment regime.

**Results:**

A total of 1020 patients were diagnosed with a clinical in situ or T1 oesophageal or gastro-oesophageal junction cancer without lymph node or distance metastasis. The proportion of patients who received endoscopic treatment increased from 2.5% in 2000 to 58.1% in 2014. During the same period the proportion of patients who received surgery decreased from 57.5 to 23.1%. Five-year relative survival of all patients was 69%. Five-year relative survival after endoscopic therapy was 83% and after surgery 80%. Relative excess risk analyses showed no significant difference in survival between patients in the endoscopic therapy group and patients in the surgery group after adjustment for age, sex, clinical TNM classification, morphology and tumour location (RER 1.15; CI 0.76–1.75; *p* 0.76).

**Conclusion:**

Our results demonstrate an increase in endoscopic treatment and a decrease of surgical treatment for in situ and T1 oesophageal/GOJ cancer between 2000–2014 in the Netherlands. The relative 5-year survival after endoscopic treatment is high (83%) and comparable with surgery (80%).

**Supplementary Information:**

The online version contains supplementary material available at 10.1007/s00464-023-09914-x.

Oesophageal cancer is the eight most common cancer worldwide and the sixth leading cause of cancer related mortality [1, 2]. The incidence of oesophageal cancer increases worldwide [1, 3, 4]. In 1913 a German surgeon named Torek, performed the first thoracic oesophagectomy for oesophageal cancer in New York City and the patient survived for 12 years [5]. Ever since, oesophagectomy has been standard of care in case of resectable oesophageal cancer, until the first endoscopic cap resection for early neoplastic lesions in the oesophagus was performed by the Japanese surgeon Inoue in 1990 [6]. Endoscopic treatment of early oesophageal cancer in the Netherlands started from the beginning of this century. Endoscopic resection is a less invasive and safe organ preserving treatment with neglectable peri-procedural mortality, low complication rates and at lower costs [7–10]. However, endoscopic resection is only possible for early-stage cancer and curative endoscopically treatable cancer is defined as mucosal and superficial submucosal cancer with a infiltration depth ≤ 500 µm. Recent studies reported a shift in preferred treatment in case of resectable oesophageal cancer, describing an increasing proportion of early oesophageal cancer treated by endoscopic therapy [11–13] with a reciprocal decrease in surgical treatments over time [12].

Previous research showed comparable survival after endoscopically or surgically treated oesophageal cancer in the United States and Asia [7, 10, 12, 14, 15]. The scientific question of the present study was how the treatment of T1 oesophageal cancer changed in the Netherlands over time and if this frame shift had influence on survival, aiming to provide insight into treatment and survival for patients with clinical T1 oesophageal and gastro-oesophageal junction (GOJ) cancer over a fifteen-year period between 2000 and 2014 in the Netherlands.

## Materials and methods

### Data collection

Data were obtained from the nationwide population-based Netherlands Cancer Registry (NCR). The NCR is based on notification of all newly diagnosed malignancies in the Netherlands by the national automated pathological archive (PALGA). Additional sources are the national registry of hospital discharge, hematology departments and radiotherapy institutions. Information on patient characteristics, diagnosis, tumour characteristics, treatment, follow-up and vital status are routinely extracted from the medical records by specially trained registrars operating on behalf of the NCR. Vital status is obtained through annual linkage with civil municipal registries and up to date until the first of February for 2019. The institutional review board approved this study.

### Patients

All patients diagnosed with clinical in situ and T1 oesophageal or GOJ cancer without lymph node or distance metastasis between 1 January 2000 and 31 December 2014 were extracted from the NCR. Primary outcome parameters defined were trends in treatment modalities over time and relative survival of each treatment regime.

Tumours were classified as adenocarcinoma, squamous cell carcinoma or other using the International Classification of Diseases for Oncology (ICD-O) morphology codes. Tumour staging was established according to the tumour-node-metastasis (TNM) classification that was valid at the time of diagnosis. High-grade dysplasia was classified as carcinoma in situ (Tis). Patients diagnosed before 2002 were staged according to the fifth edition, patients diagnosed between 2003 and 2009 according to the sixth edition and patients diagnosed between 2010 and 2014 according to the seventh edition. For an adequate comparison of clinical tumour stages all TNM stages were converted to the TNM 6 classification. There was no conversion necessary to transform TNM 5 into TNM 6. Data classified with TNM 7 had to be converted into TNM 6, which includes the recoding of cT1a and cT1b into cT1 and cN1, N2 and N3 into cN1. After a change in coding regulations of the NCR regarding distant metastasis (cM) in 2010, fewer diagnostic procedures were required to register a cM0 or cM1. After 2010 no cMx was reported in our dataset. Based on previous analyses of these data we assume that patients with a cMx before 2010 were most likely considered as cM0 in clinical practice. This was the reason to include 49 patients with unknown distance metastasis and integrate them as cM0 for the analyses. Treatment was classified as endoscopic therapy, surgery (with or without neoadjuvant CRT), no treatment (including active surveillance) or other treatment. Other treatment included treatments as chemotherapy, radiotherapy, (definitive) CRT, unknown type of palliative therapy or symptomatic treatment with an endoprothesis. A total of 68 patients who first received endoscopic treatment and surgery thereafter were classified into the group of surgical therapy. Although the exact indications for adjuvant surgery were not registered in the national database we assume that there was a R1 resection, pathological T1 cancer with high risk stigmata defined as deep submucosal or lymphovascular invasion, or a more advanced pathological T stage.

### Statistical analysis

For descriptive statistics mean and standard deviation were used in case of normally distributed variables. Groups were compared using the unpaired *t*-test. In case of a skewed distribution, median and range were used and independent groups were compared with the Mann Whitney *U* test. Categorical data were expressed as percentages and groups were compared using the Chi-square or the Fisher’s exact test, when appropriate. We used the relative survival as the best approximation of the cancer specific survival. Relative survival can be defined as the ratio of observed survival in the selected compared to the expected survival of the corresponding general population based on age, gender and year. Relative survival was calculated using the Pohar Perme method [16]. Relative excess risk analyses were performed to study independent association of baseline variables as a factor affecting relative survival and were reported as relative excess risk (RER) and 95% CI. All data analyses were performed by using SPSS 25 Software for Windows and Stata (14.2) and reported *p* values of ≤ 0.05 were considered statistically significant.

## Results

**Fig. 1:**
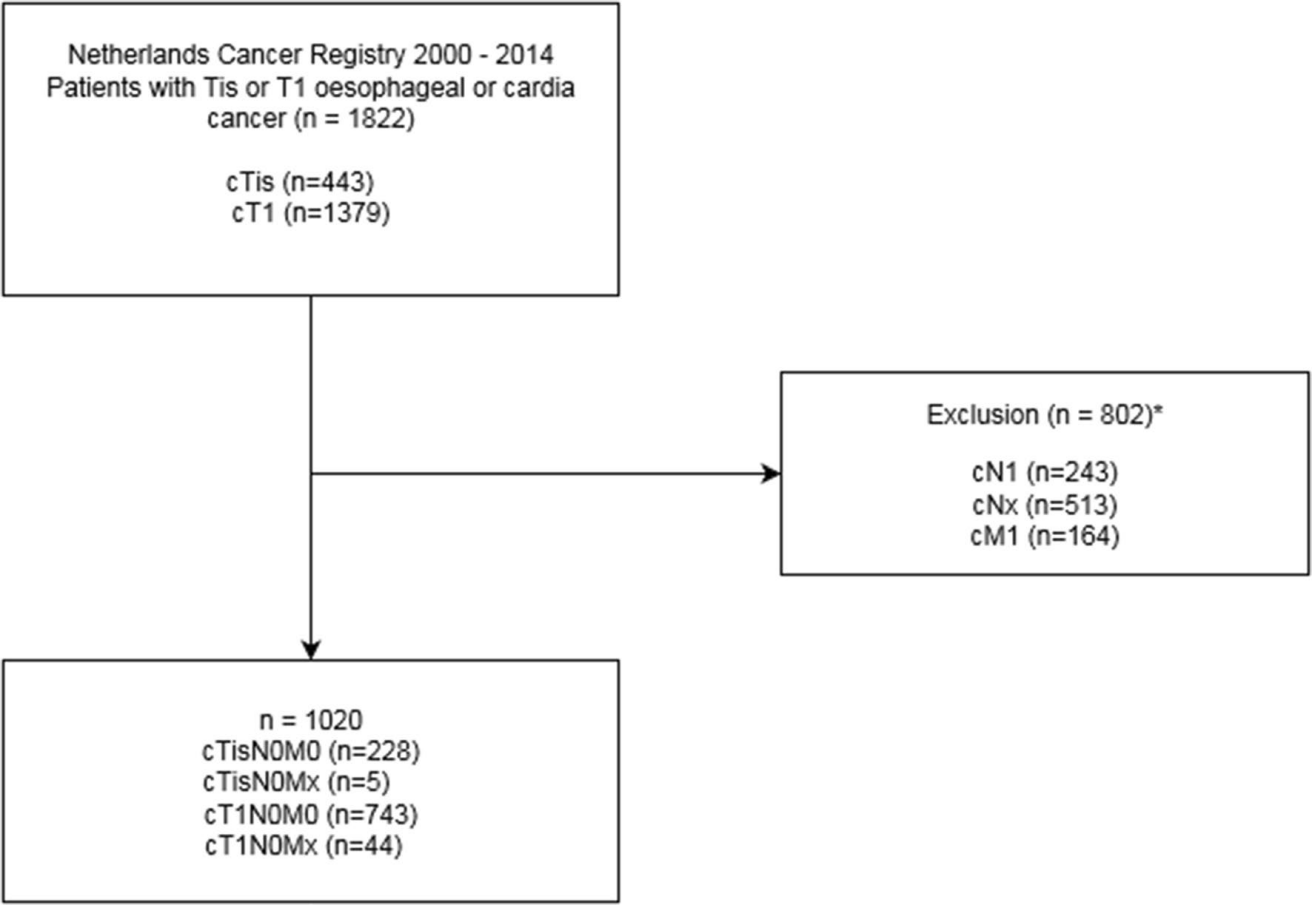
Study flowchart of patients diagnosed with clinical in situ and T1 oesophageal or GOJ cancer between 1 January 2000 and 31 December 2014, along with the final 1020 included patients diagnosed with clinical in situ and T1 oesophageal or GOJ cancer without lymph node or distance metastasis

A total of 1822 patients were diagnosed with a clinical in situ or T1 oesophageal or GOJ cancer between January 2000 and December 2014 in the Netherlands. Patients with metastatic disease (*n* = 285) and positive or unknown lymph node metastasis (*n* = 513) were excluded (Fig. 1).

Detailed patient, tumour and therapy characteristics of the remaining 1020 patients with a clinical in situ or T1 oesophageal carcinoma are shown in Table 1, 2, 3. Overall, almost half of the patients underwent surgery (41.5%), a little less received endoscopic therapy (38.2%) and around twenty per cent of patients underwent other treatment (12.3%) or no treatment et al. (7.7%). The majority of patients were male (73%) and had an adenocarcinoma (77.5%). Most patients were clinically diagnosed with a T1 tumour (77.2%). An overall rise in the incidence of clinical in situ and T1 oesophageal carcinoma was observed between 2000 and 2014.

**Table 1:** Baseline patient characteristics of all patients with clinical in situ and T1 oesophageal or GOJ cancer without lymph node or distance metastasis (*n* = 1020)

	Endoscopic therapy *n* = 390 (%)	Surgery *n* = 426 (%)	*p* value	Other *n* = 125 (%)	No therapy *n* = 79 (%)	All patients *n* = 1020 (%)
Age			< 0.01			
< 60 years	96 (24.6)	141 (33.1)		14 (11.2)	4 (5.1)	255 (25.0)
60–70 years	124 (31.8)	167 (39.2)		26 (20.8)	15 (19.0)	332 (32.5)
70–80 years	116 (29.7)	101 (23.7)		45 (34.4)	29 (36.7)	289 (28.3)
> 80 years	54 (13.8)	17 (4.0)		42 (33.6)	31 (39.2)	144 (14.1)
Gender			0.042			
Male	306 (78.5)	308 (72.3)		80 (64.0)	55 (69.6)	749 (73.4)
Female	84 (21.5)	118 (27.7)		45 (36.0)	24 (30.4)	271 (26.6)

**Table 2:** Baseline tumour characteristics of all patients with clinical in situ and T1 oesophageal or GOJ cancer without lymph node or distance metastasis (*n* = 1020)

	Endoscopic therapy *n* = 390 (%)	Surgery *n* = 426 (%)	*p* value	Other *n* = 125 (%)		No therapy *n* = 79 (%)	All patients *n* = 1020 (%)
Tumour location			< 0.001				
Oesophagus	376 (96.4)	349 (81.9)		116 (92.0)		59 (74.7)	899 (88.1)
GOJ	14 (3.6)	77 (18.1)		10 (8.0)		20 (25.3)	121 (11.9)
Morphology			0.676				
Squamous cell carcinoma	57 (14.6)	71 (16.7)		48 (38.4)		14 (17.7)	190 (18.6)
Adenocarcinoma	319 (81.8)	342 (80.3)		72 (57.6)		57 (72.2)	790 (77.5)
Unknown	14 (3.6)	13 (3.1)		5 (4.0)		8 (10.1)	40 (3.9)
Differentiation grade			< 0.001				
Good	46 (11.8)	46 (10.8)		2 (1.6)		3 (3.8)	97 (9.5)
Normal	110 (28.2)	141 (33.1)		19 (15.2)		14 (17.7)	284 (27.8)
Bad	43 (11.0)	116 (27.2)		25 (20.0)		7 (8.9)	191 (18.7)
Undifferentiated	1 (0.3)	0 (0)		0 (0)		2 (2.5)	3 (0.3)
Unknown	190 (48.7)	123 (28.9)		79 (63.2)		53 (67.1)	445 (43.6)
cTNM classification			< 0.001				
cTisN0M0	135 (34.6)	48 (11.3)		17 (13.6)		33 (41.8)	233 (22.8)
cT1N0M0	255 (65.4)	378 (88.7)		108 (86.4)	46 (58.2)		787 (77.2)
(y)pTNM classification							
(y)pT			< 0.001				
pT0	0 (0)	8 (1.9)		n/a	n/a		8 (1.0)
pTis	70 (17.9)	29 (6.8)		n/a	n/a		99 (12.1)
pT1	233 (59.7)	309 (72.5)		n/a	n/a		542 (66.4)
pT2	4 (1.0)	42 (9.9)		n/a	n/a		46 (5.6)
pT3	0 (0)	25 (5.9)		n/a	n/a		25 (3.1)
pTx	83 (21.3)	13 (3.1)		n/a	n/a		96 (11.8)
(y)pN			< 0.001				
pN0	61 (15.6)	302 (70.9)		n/a	n/a		363 (44.5)
pN +	1 (0.3)	64 (15)		n/a	n/a		65 (8.0)
pNx	328 (84.1)	60 (14.1)		n/a	n/a		388 (47.5)
(y)pM			< 0.001				
pM0	105 (26.9)	232 (54.5)		n/a	n/a		337 (41.3)
pM +	0 (0)	3 (0.7)		n/a	n/a		3 (0.4)
pMx	285 (73.1)	191 (44.8)		n/a	n/a		476 (58.3)

**Table 3:** Baseline therapy characteristics of all patients with clinical in situ and T1 oesophageal or GOJ cancer without lymph node or distance metastasis (*n* = 1020)

	Endoscopic therapy *n* = 458 (%)	Surgery *n* = 358 (%)	*p* value	Other *n* = 125 (%)	No therapy *n* = 79 (%)	All patients *n* = 1020 (%)
Additional RCT			0.040			
Neo-adjuvant	0 (0)	27 (5.4)		n/a	n/a	27 (3.3)
Adjuvant	14 (3.6)	2 (0.4)		n/a	n/a	16 (2.0)
Additional surgery after endoscopic therapy						
Yes	n/a	68 (14.8)		n/a	n/a	68 (8.3)
Period of diagnosis			< 0.001			
2000–2004	25 (6.4)	107 (25.1)		44 (35.2)	19 (24.1)	195 (19.1)
2005–2009	112 (28.7)	142 (33.3)		29 (23.2)	20 (25.3)	303 (29.7)
2010–2014	253 (64.9)	177 (41.5)		54 (41.6)	40 (50.6)	522 (51.2)

### Endoscopic therapy vs. surgery

Patients who underwent surgery were significantly younger than patients treated with endoscopic therapy (median 64 vs. 68 years; *p* < 0.001) and the group with endoscopic therapy significantly more often had a clinical in situ tumour (*p* < 0.001). No significant difference was observed with respect to morphology. Adjuvant radiotherapy, chemotherapy or CRT after initial endoscopic treatment was administered in 14 (3.3%) patients (Table 1, 2, 3).

### Change in treatment modalities

**Fig. 2:**
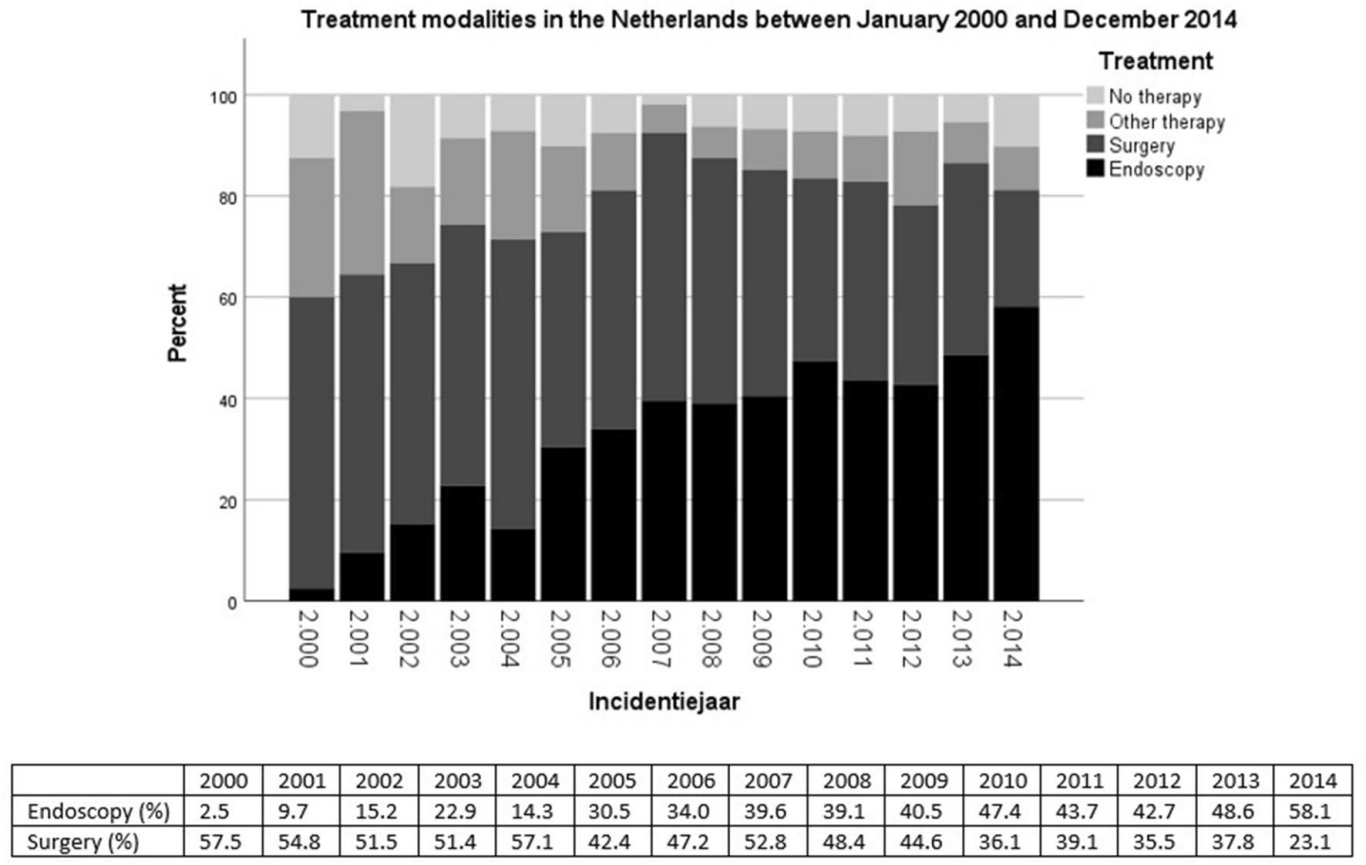
Percentage of patients divided in treatment modalities in the Netherlands between January 2000 and December 2014

The proportion of patients who received endoscopic treatment increased from 2.5% in 2000 to 58.1%in 2014. During the same period the proportion of patients who received surgery decreased from 57.5% in 2000 to 23.1% in 2014. Figure 2 shows the use of treatment modalities for all patients between 2000 and 2014.

### Survival

**Fig. 3:**
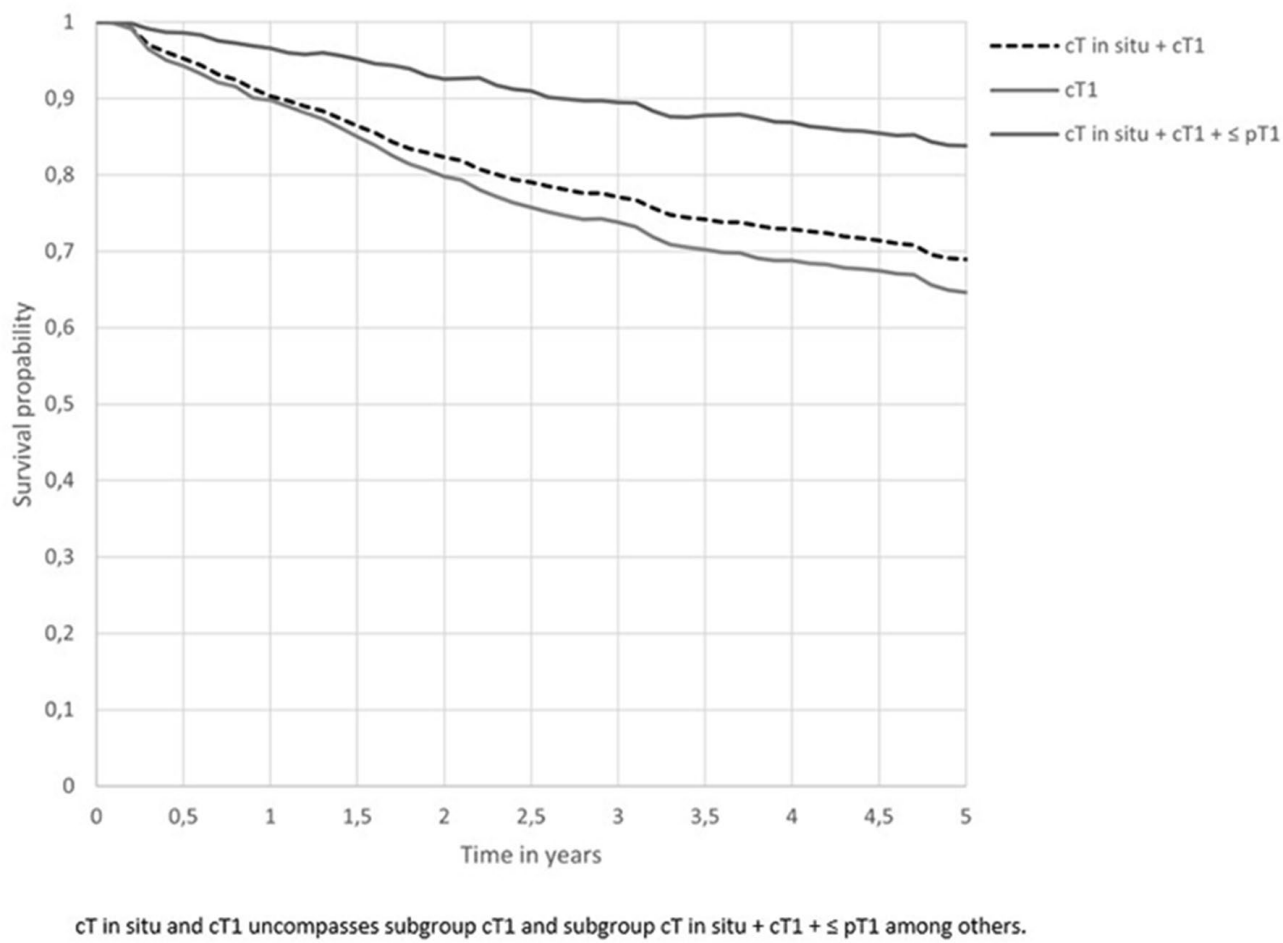
5-year relative survival

**Fig. 4:**
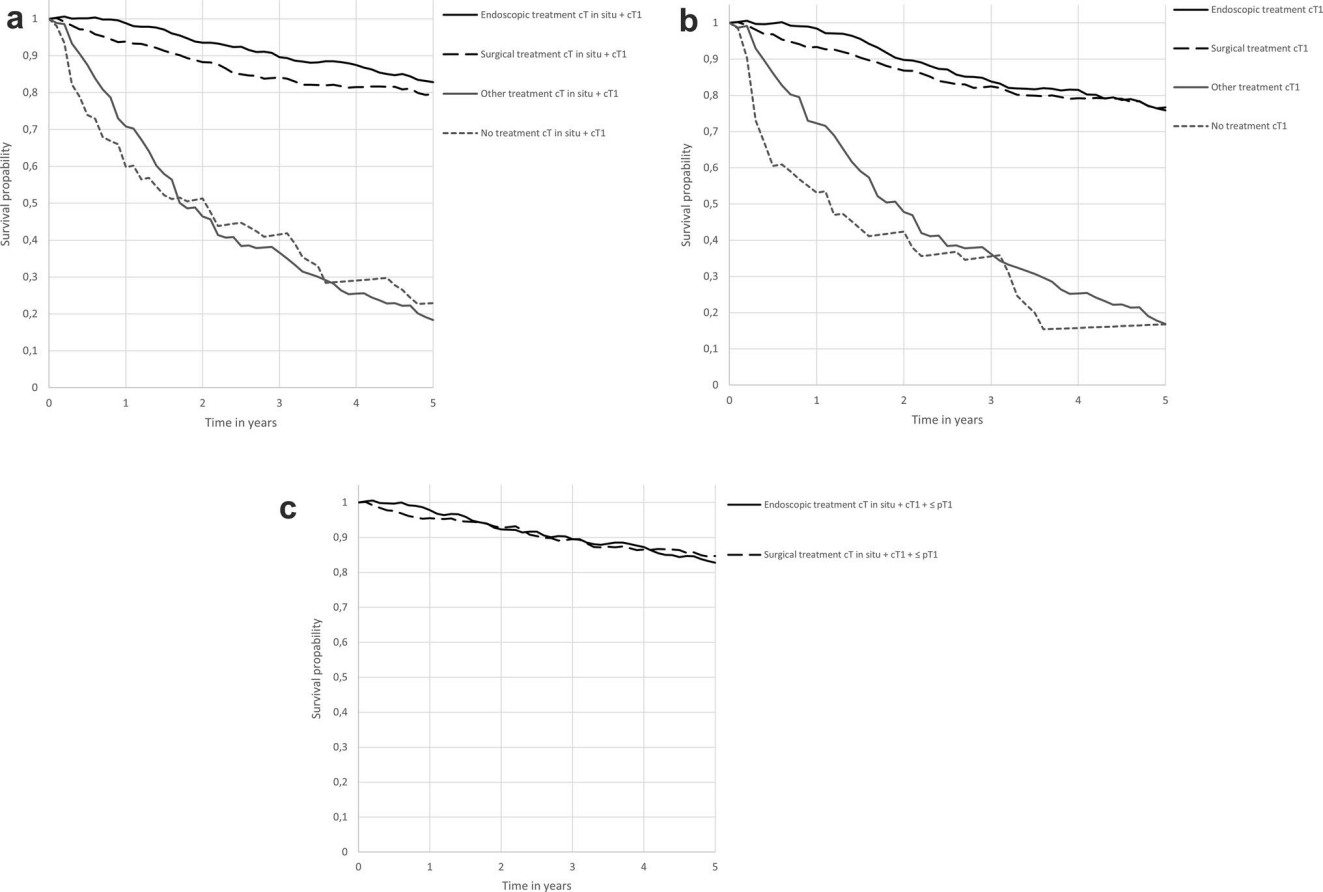
**a** 5-year relative survival of patients with clinical in situ and clinical T1 oesophageal or GOJ cancer without lymph node or distance metastasis per treatment regimen. **b** 5-year relative survival of patients with clinical T1 oesophageal or GOJ cancer without lymph node or distance metastasis per treatment regimen. **c** 5-year relative survival of patients with clinical in situ and clinical T1 and pathological ≤ T1 oesophageal or GOJ cancer without lymph node or distance metastasis per treatment regimen

Five-year relative survival of all patients with clinical in situ or T1 oesophageal or GOJ cancer was 69% (Fig. 3). Relative survival per treatment modality is shown in Fig. 4a. Five-year relative survival after endoscopic therapy was 83%, after surgery 80%, after other treatment 27% and with no treatment 19%. Relative excess risk analyses showed no significant difference in survival between patients in the endoscopic therapy group and patients in the surgery group after adjustment for age, sex, clinical TNM classification, morphology and tumour location (RER 1.15; CI 0.76–1.75; *p* = 0.76) (Table 4).

**Table 4:** Relative survival for patients treated with endoscopic therapy or surgery

5-year relative survival	5-year relative survival (%)	RER (95% CI)	*p*-value
All patients			
Endoscopic therapy	83%	1.00 (referent)	
Surgery	80%	1.15 (0.76–1.75)	0.76
Subgroup (cT1)			
Endoscopic therapy	76%	1.00 (referent)	
Surgery	77%	1.15 (0.76–1.75)	0.50
Subgroup (cT in situ + cT1 + ≤ pT1			
Endoscopic therapy	83%	1.00 (referent)	
Surgery	85%	0.82 (0.50–1.33)	0.42

*CI* confidence interval, *RER* relative excess risk

RER after adjustment for age, sex, clinical TNM classification, morphology and tumour location

### Subgroup analysis for patients with clinical T1 oesophageal or GOJ cancer

A total of 787 (77.2%) patients were diagnosed with a clinical T1 oesophageal or GOJ cancer without lymph node or distance metastasis. Around 50% of the patients received endoscopic therapy (48.0%) or underwent surgery (32.4%). Almost twenty percent underwent other treatment (13.7%) or no treatment at all (5.8%). In a subgroup analysis, 5-year relative survival of all patients with clinical T1 oesophageal or GOJ cancer was 65% (Fig. 3). Five-year relative survival after endoscopic therapy was 76%, 77% after surgery, 18% after other treatment and 19% with no treatment as shown in Fig. 4b. Relative excess risk analyses for the subgroup T1 oesophageal or GOJ cancer showed no significant differences in survival between patients in the endoscopic therapy group and patients in the surgery group after adjustment for age, sex, clinical TNM classification, morphology and tumour location (RER 1.15; CI 0.76–1.75; *p* = 0.50) (Table 4).

### Subgroup analysis for patients with clinical in situ or T1 and a pathological ≤ T1 stage oesophageal or GOJ cancer

A total of 649 (63.6%) patients were diagnosed with a clinical in situ or T1 and a pathological ≤ T1 stage oesophageal or GOJ cancer without lymph node or distance metastasis. Almost half of the patients received endoscopic therapy (47.3%) or underwent surgery (52.7%). In a subgroup analysis, 5-year relative survival of all patients with clinical in situ or T1 and a pathological ≤ T1 stage oesophageal or GOJ cancer was 84% (Fig. 3). Five-year relative survival after endoscopic therapy was 83% and 85% after surgery as shown in Fig. 4c. Relative excess risk analyses for the subgroup with a clinical in situ or T1 and a pathological T1 oesophageal or GOJ cancer showed no significant differences in survival between patients in the endoscopic therapy group and patients in the surgery group after adjustment for age, sex, clinical TNM classification, morphology and tumour location (RER 0.82; CI 0.50–1.33; *p* = 0.42) (Table 4).

## Discussion

This nationwide population-based study assess insight into treatment and survival for patients with clinical T1 oesophageal and GOJ cancer over a fifteen-year period between 2000 and 2014 in the Netherlands. The results of our study demonstrate an increase in patients diagnosed with in situ and T1 oesophageal/ GOJ cancer and shows a frame shift in treatment modalities over the defined study period. An increase in endoscopic resections and a concomitant decrease in surgical treatments over time were demonstrated.

These results confirm the increasing proportion of early oesophageal cancer treated by endoscopic therapy in literature [11–13]. Previous studies reporting this topic from the USA demonstrated similar increasing percentages for endoscopic resection from 2.8–19.0% to 24.1–87.7% over time of almost 24 years, respectively [11–13, 17]. A decrease in surgical resections from 87.2% to 69.2% was reported over the time period of 20 years, respectively [12].

In the Netherlands, it is expected that the increase in endoscopic resections is due to the increasing possibilities of new endoscopic resection techniques in combination with the centralisation of expertise in Barrett’s oesophagus treatment. This latter factor has especially contributed to an increasing expertise in endoscopic resection of early carcinomas [8, 18–21].

The relative 5-year survival was high in the endoscopic treatment group (83%) and comparable with the surgical group (80%) after adjustment for age, sex, clinical TNM classification, morphology and tumour location. However, these results must be interpreted with caution. There is an observed difference in a more advanced stage of oesophageal or GOJ cancer ((y)pT2 and (y)pT3) after final pathology in the surgery group (15.7%), compared to the endoscopic resection group (1.0%). It seems possible that endoscopic misclassifying depth of invasion (T stage) might have occurred, or it could be attributed to selection. Detailed information about specific tumour characteristics such as tumour depth and lymphovascular invasion or patient characteristics such as performance status or ASA score, were missing in this cancer registry. Therefore, it remains unclear whether or not patients in both groups were similar and the difference in survival between the endoscopic and surgically treated group might be explained by selection bias. In subgroup analysis we therefore decided to compare clinical in situ or T1 cancer with the same pathological T stage (≤ pT1). The relative 5-year survival was high in the endoscopic group (83%), but not significantly different when compared to the surgical group (85%) after adjustment for age, sex, clinical TNM classification, morphology and tumour location. The findings from this study suggest that endoscopic treatment for clinical T1 oesophageal and GOJ cancer is an alternative therapy with a comparable relative 5-year survival.

Explaining the observed small increase in survival over time is merely hypothetical. The development of new endoscopic diagnostic tools and thus better staging and selection, and endoscopic resection techniques, centralisation of oesophageal cancer treatment, as well as the centralisation of endoscopic treatments might have contributed to the observed improved survival [22–24].

In patients with no treatment after diagnosis we found a relative 5-year survival rate of 19%, which appear to be quite low for early-stage tumours. It should be mentioned that more than 75% of the patients in this group were diagnosed at age above 70. Around 40% of the patients in this group were diagnosed with a cTisN0M0 carcinoma. A possible explanation for this observation could be that these patients had severe comorbidity, that was more life-threatening than the cTis/cT1N0M0 esophageal cancer. Unfortunately we could not retrieve information on comorbidity from this data registry.

To our knowledge, this is the first study demonstrating the survival after endoscopic resection and or surgery of early oesophageal cancer in Europe. When comparing our results to those of the older studies, a study using a hospital-based cancer registry in the USA reported a comparable different 5-year survival rates between endoscopically or surgically treated T1 oesophageal cancer (87.6% vs 76.5%). Our subgroup analysis, comparing endoscopic treatment with surgery for T1 oesophageal and GOJ cancer without lymph node or distance metastasis (76% vs 77%), is lower for the endoscopic treatment group when compared with the 5-year oesophageal cancer specific survival for T1N0M0 oesophageal cancer in the USA(81.7% vs 75.8%) [11]. However, that study included only patients with oesophageal T1N0M0 cancer, not mentioning whether this was the clinical or the post-procedural pathological TNM stage.

There is an ongoing discussion in literature about superiority in surgery (esophagectomy versus a gastrectomy) in case of GOJ cancer in effecting overall survival. In a nationwide cohort study, Köeter et al. showed no difference in the overall survival between esophagectomy or gastrectomy for GOJ cancer in the Netherlands [25]. Due to the comparable resection technique and morbidity between distal esophageal and GOJ cancer, we included GOJ cancers in our study. International data to confirm our national data is still being collected in the international CARDIA trial exploring the best surgical strategy for GOJ cancer [26]. This study confirms previous literature showing an increasing incidence of oesophageal cancer [1, 3, 4]. The increasing incidence might reasonably contribute to an increase in healthcare cost. A recent study calculated a four time higher minimum cost associated with surgery compared to endoscopic treatment [10]. Therefore, an endoscopic evaluation to assess the possibility of endoscopic resection in every patient with an early oesophageal or GOJ cancer could potentially reduce healthcare costs.

The strength of this study is the large study population with real life data on treatment modalities and survival of 15 years in the Netherlands. This study has also some limitations. First, data recorded by NCR does not allow evaluation on comorbidities and specific histopathologic data such as tumour depth and lymphovascular invasion. No individual information on clinical decision making for the choice of treatment regimen is available. Second, there might be a possible underestimation of endoscopically treated patients in the earlier period in the registry. Third, tumour staging was converted into the TNM 6 classification due to two times a change in TNM classification between 2000 and 2014. As a result, the small number of patients in the subgroups cT1a (105 patients) and cT1b (74 patients) limits our possibility for subgroup survival analyses. For further research the survival after endoscopic resection or surgery stratified for cT1a and cT1b after 2010 is ongoing. The T1b group might harbour a subgroup of patients who could also have benefit from an endoscopic resection.

## Conclusion

Our results demonstrate an increase in the use of endoscopic treatment and a decrease of surgical treatment for in situ and T1 oesophageal/GOJ cancer between 2000–2014 in the Netherlands. The relative 5-year survival after endoscopic treatment was high (83%) and comparable to surgery (80%).

## Supplementary Information

Below is the link to the electronic supplementary material.

Supplementary file1 (TIF 436 KB) 5-year overall survival of patients with clinical T1 oesophageal or GOJ cancer without lymph node or distance metastasis per treatment regimen

Supplementary file2 (TIF 439 KB) 5-year overall survival of patients with clinical in situ and clinical T1 oesophageal or GOJ cancer without lymph node or distance metastasis per treatment regimen

Supplementary file3 (TIF 389 KB) 5-year overall survival of patients with clinical in situ and clinical T1 and pathological ≤ T1 oesophageal or GOJ cancer without lymph node or distance metastasis per treatment regimen

Supplementary file4 (JPG 50 KB) 5-year overall survival
