# Supplementary figures and images for: Endoscopic therapy replaces surgery for clinical T1 oesophageal cancer in the Netherlands: a nationwide population-based study
Source: Surg Endosc. 2023 Feb 27;37(6):4535–44. doi: 10.1007/s00464-023-09914-x (PMC10234922; doi:10.1007/s00464-023-09914-x)

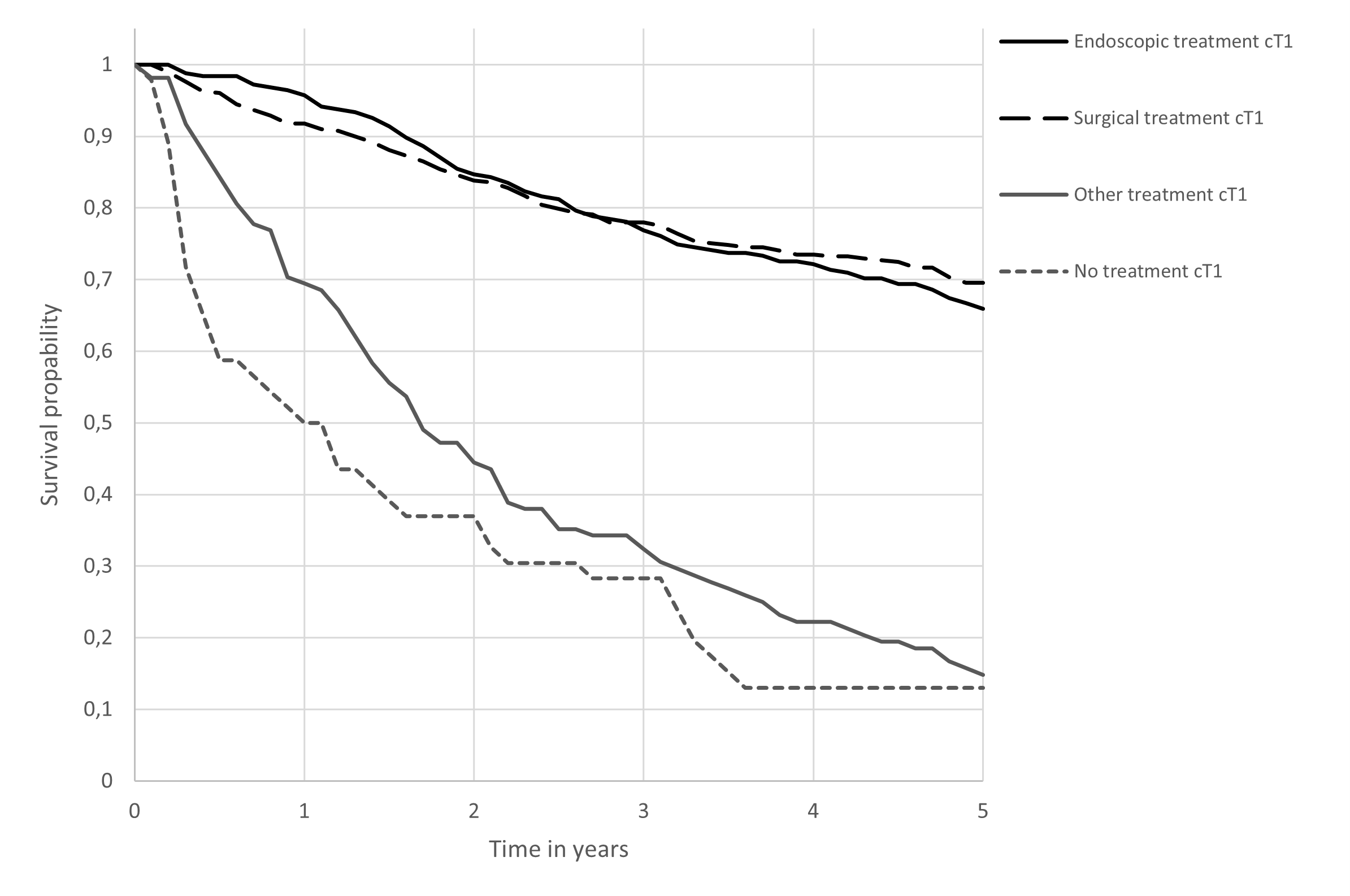

Supplement: Supplementary file 1 — Supplementary file1 (TIF 436 KB) 5-year overall survival of patients with clinical T1 oesophageal or GOJ cancer without lymph node or distance metastasis per treatment regimen [file 464_2023_9914_MOESM1_ESM.tif]

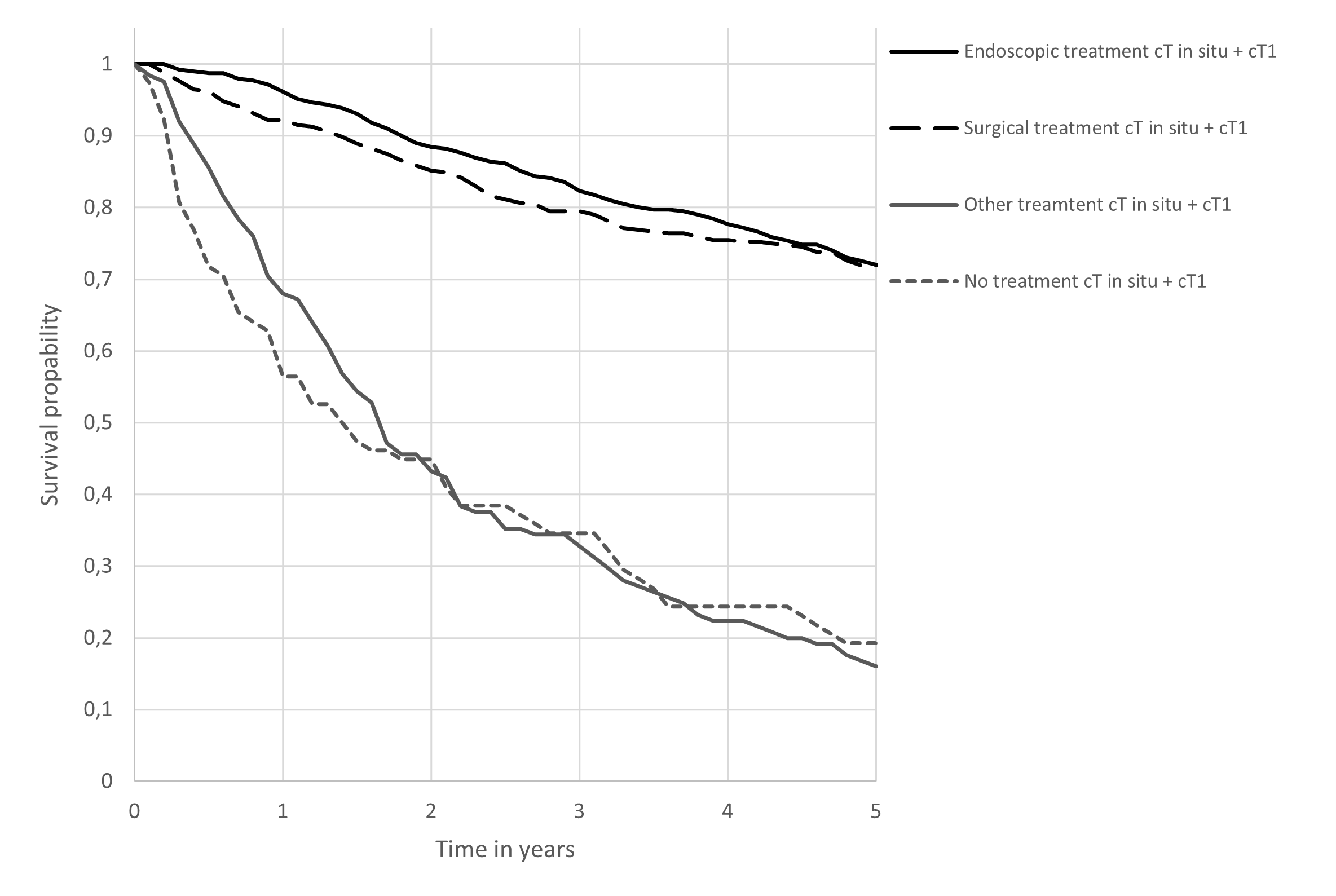

Supplement: Supplementary file 2 — Supplementary file2 (TIF 439 KB) 5-year overall survival of patients with clinical in situ and clinical T1 oesophageal or GOJ cancer without lymph node or distance metastasis per treatment regimen [file 464_2023_9914_MOESM2_ESM.tif]

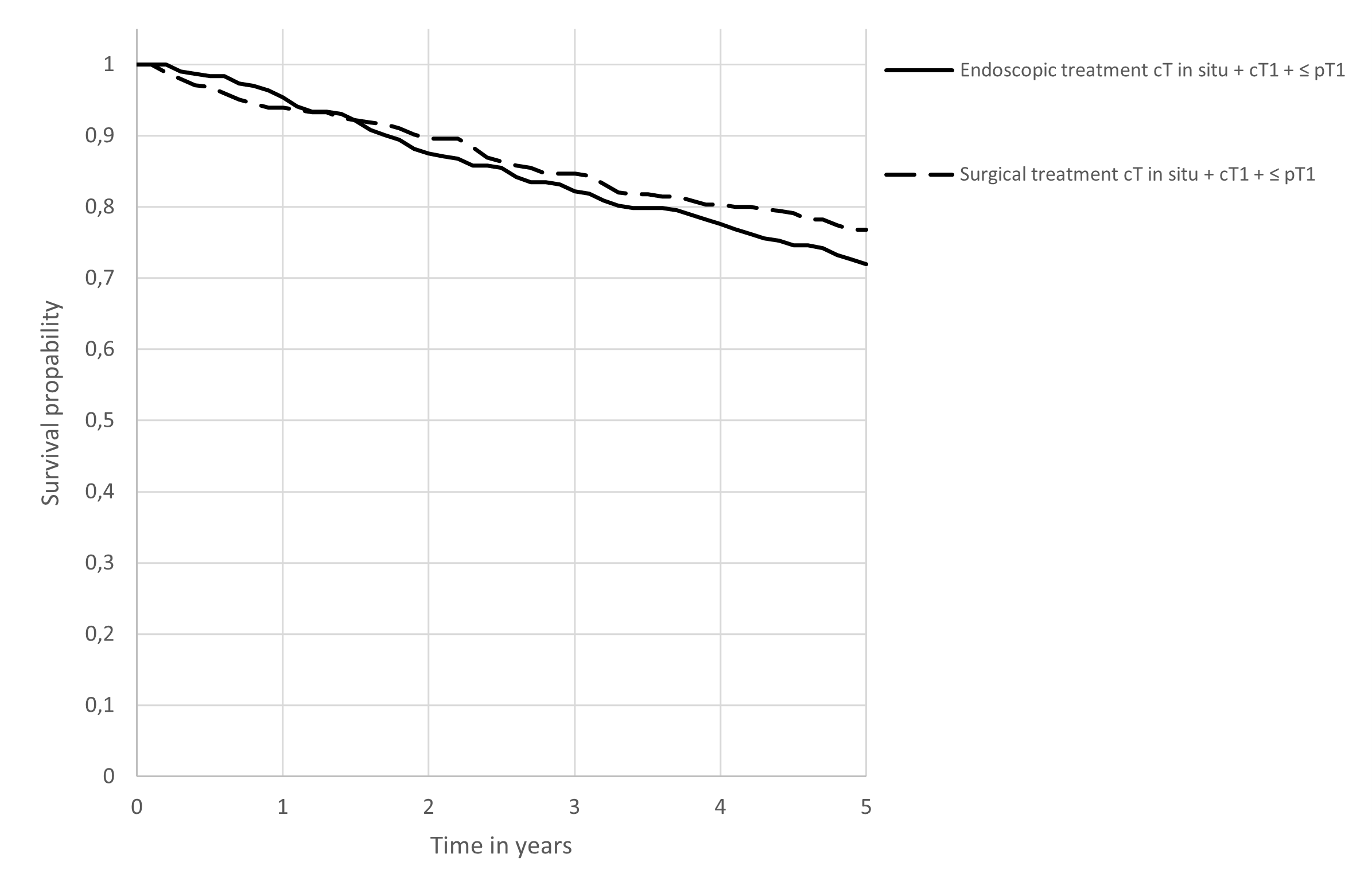

Supplement: Supplementary file 3 — Supplementary file3 (TIF 389 KB) 5-year overall survival of patients with clinical in situ and clinical T1 and pathological ≤ T1 oesophageal or GOJ cancer without lymph node or distance metastasis per treatment regimen [file 464_2023_9914_MOESM3_ESM.tif]

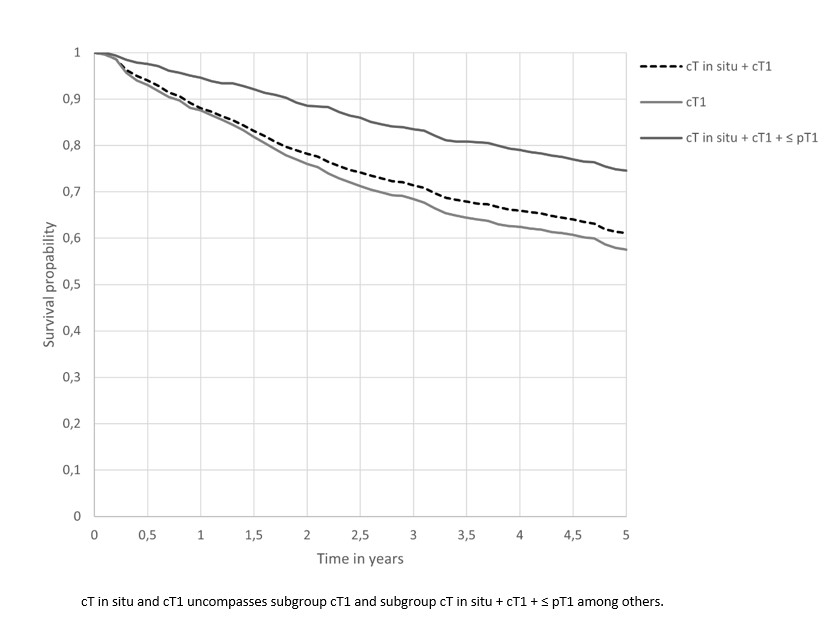

Supplement: Supplementary file 4 — Supplementary file4 (JPG 50 KB) 5-year overall survival [file 464_2023_9914_MOESM4_ESM.jpg]
